# Supplementary material for: Intellectual disability literacy and its connection to stigma: A multinational comparison study in three European countries
Source: PLoS One. 2020 Oct 15;15(10):e0239936. doi: 10.1371/journal.pone.0239936 (PMC7561148; doi:10.1371/journal.pone.0239936)
Supplement: S1 File — (PDF) [file pone.0239936.s001.pdf]

Labelled vignettes of the *Intellectual Disability Literacy Scale* (IDLS)

English-language:

James has a learning disability (mental handicap).<br>He is 22 and lives at home with his parents and younger brother. He found school a struggle and left without any qualifications. He has had occasional casual jobs since. When his parents try to encourage him to make plans for his future, James has few ideas or expresses ambitions that are well out of his reach. Rather than having him at home doing nothing, his mum has been trying to teach James new skills, such as cooking a meal, but James has struggled to follow her instructions. He opened up a bank account with his parents' help, but has little idea of budgeting and, unless his parents stop him, will spend all his benefits on comics and DVDs as soon as he receives his money.

German-language:

Johannes hat eine intellektuelle Behinderung (geistige Behinderung).<br>Er ist 22 Jahre alt und wohnt mit seinem jüngeren Bruder zuhause bei seinen Eltern. In der Schule hatte er Schwierigkeiten und verließ sie ohne Abschluss. Seitdem hat er ein paar Gelegenheitsjobs gehabt. Wenn seine Eltern ihn dazu bewegen wollen über seine Zukunft nachzudenken, hat Johannes wenig konkrete Vorstellungen und äußert Ziele, die für ihn unerreichbar sind. Um ihn zuhause zu beschäftigen, versucht Johannes Mutter ihm nützliche Fähigkeiten wie zum Beispiel Kochen beizubringen, aber er hat Probleme, ihren Anweisungen zu folgen. Mit Hilfe seiner Eltern hat er ein Bankkonto eröffnet, aber versteht es nicht mit Geld zu haushalten. Wenn seine Eltern ihn nicht bremsen, gibt Johannes all seine staatlichen Zuschüsse für Comics und DVDs aus.
